# Complete genome sequence of a virulent *Klebsiella pneumoniae* bacteriophage KPP-X1 harboring 26 tRNA genes

**DOI:** 10.1128/mra.01062-25

**Published:** 2026-02-27

**Authors:** Xi Wu, Yuhui Yang, Jingmei Ding, Wanbing Liu, Mengyu Shen

**Affiliations:** 1 Basic Medical Laboratory, General Hospital of Central Theater Command 691967 , Wuhan, China; 2 Hubei Key Laboratory of Central Nervous System Tumour and Intervention, Wuhan, China; 3 School of Nursing, Army Medical University, Chongqing, China; 4 Department of Outpatient, General Hospital of Central Theater Command 691967 , Wuhan, Hubei, China; 5 Department of Transfusion, General Hospital of Central Theater Command 691967 , Wuhan, Hubei Province, China; Loyola University Chicago, Chicago, Illinois, USA

**Keywords:** bacteriophage, *Klebsiella pneumoniae*, tRNA, genomic analysis

## Abstract

KPP-X1, a new virulent *Klebsiella pneumoniae* bacteriophage, was isolated from sewage samples and sequenced. The 146,898-bp genome has 251 predicted genes. The genome of KPP-X1 does not contain lysogeny-related elements, resistance genes, or virulence factors and encodes abundant tRNAs, making it a promising candidate for phage therapy.

## ANNOUNCEMENT

*Klebsiella pneumoniae* is one of the most common causes of nosocomial infections (1, 2). The antibiotic resistance of *Klebsiella pneumoniae*, which arises from mechanisms like β-lactamase production, efflux pump overexpression, and biofilm formation, poses significant treatment challenges (3). To advance phage therapy for drug-resistant *Klebsiella pneumoniae* infections, we herein present the complete genome sequence of a new bacteriophage, KPP-X1.

**Fig 1:**
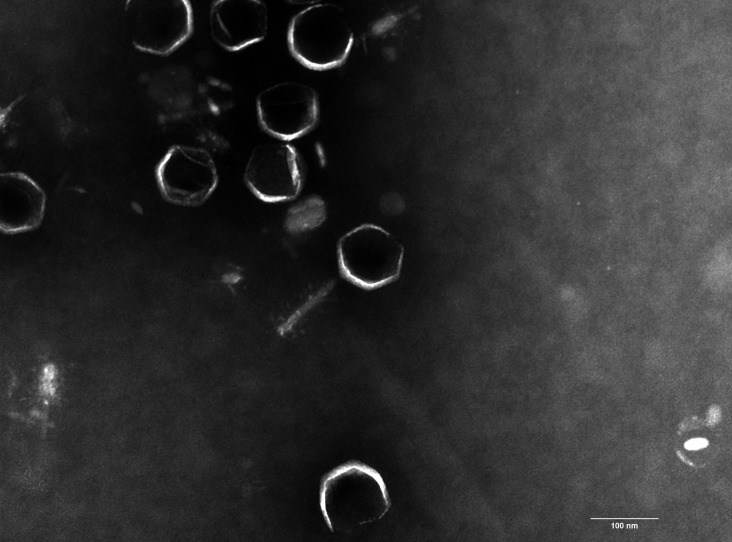
Transmission electron micrograph of *Klebsiella pneumoniae* phage KPP-X1, visualized using a Hitachi HT7800 microscope at 80 kV and 50,000× magnification. The sample was negatively stained with 2% phosphotungstic acid. Scale bar, 200 nm.

Virulent phage KPP-X1 was isolated from hospital sewage (Wuluo Road 627, Wuhan, Hubei, China) via the double-layer agar method with *Klebsiella pneumoniae* ATCC700603 as the host strain in 2022 (4, 5). Briefly, the sewage sample was centrifuged and filtered using a 0.22-µm filter. Overnight cultures of *Klebsiella pneumoniae* ATCC700603 were inoculated with filtrate at 37℃ overnight. The resulting cultures were filtered, incubated with the log-phase host bacterium (grown in Luria–Bertani [LB] liquid medium at 37℃ for ~ 3 h) for 15 min at room temperature, and then mixed with molten top agar (0.6% [wt/vol] agar). The plates were incubated at 37°C. A single plaque was picked and resuspended in LB medium for subsequent purification. KPP-X1 was purified through 3 consecutive rounds of single-plaque isolation. Transmission electron microscopy (TEM) revealed that KPP-X1 possessed an icosahedral head with an apex diameter of ~100 nm and a contractile tail; the tail measured approximately 111 nm in its noncontracted state and the contracted state shortening to ~ 50 nm (Fig. 1). The TEM suggests that KPP-X1 has a myovirus-like morphology.

Genomic DNA was extracted from high titer stocks (>10^7^ PFU/mL) using the proteinase K-SDS method (6). Briefly, phage lysates were treated with DNase I and RNase A to eliminate contaminating bacterial DNA. Proteinase K and SDS were then added to degrade phage capsid proteins; subsequently, isopropanol was introduced to precipitate phage DNA. Sequencing libraries were constructed using the Nextera DNA Flex Library Prep Kit (Illumina). Paired-end sequencing (150 bp read length) was performed on an Illumina NovaSeq 6000 platform, yielding 8,806,544 reads (coverage ~8,522× ). Raw reads were quality-filtered, and adapters were trimmed using Trimmomatic v0.39 with the following parameters: ILLUMINACLIP:TruSeq3-PE.fa:2:30:10 TRAILING:3 SLIDINGWINDOW:4:15 MINLEN:50 (7), and 8,351,304 clean reads were retained. *De novo* assembly was performed using IDBA v1.1.3 (parameters: --step 20 --mink 40) (8). The completeness assessment workflow incorporating CheckV v0.8.1 within the PhageScope was employed to verify genome completeness (9, 10). The genome was annotated using Bakta v1.5.1 (11). The predicted protein sequences were analyzed for homology to virulence factors (VFDB SetA) and antibiotic resistance genes (CARD v3.2.8) using DIAMOND v2.0.9.147 (e-value ≤1e-5, identity ≥80%) (12–14). The termini of KPP-X1 were analyzed by PhageTerm (15). Phylogenetic analysis was performed with TaxMyPhage (16).

The characteristics of KPP-X1 are summarized in Table 1. Additionally, 26 tRNA genes were detected, which correspond to 18 amino acids (excluding alanine and valine). Phages carrying abundant tRNAs exhibit enhanced infection efficiency by compensating for host tRNA depletion during late-stage replication (17). Furthermore, certain tRNA-containing phages of the *Myoviridae* family may exhibit a broader host range (18). No genes encoding known lysogeny-related elements, virulence factors, or antibiotic resistance determinants were identified. In summary, KPP-X1 can be a potential phage therapy candidate, and further identification is warranted.

**TABLE 1:** Characteristics of *Klebsiella pneumoniae* phage KPP-X1

Genome	Genome size (bp)	CheckV completeness	GC content (%)	No. of ORFs	Genome type	Taxonomic identification	Termini type	Closest relative phage (GenBank accession no.)	Nucleotide identity (%)	Query coverage (%)
KPP-X1	146,898	100	44.48	251	Linear double-stranded DNA	Mydovirus genus	Direct terminal repeats (DTR)	NC_048873.1	96.79%	86

## Data Availability

The complete genome sequence of KPP-X1 is available on GenBank, under accession number PX283063.1. BioSample and BioProject accession numbers are SAMN51607403 and PRJNA1331516. Raw sequencing data are available in the SRA under accession number SRR35566334.
